# Spatial Landslide Susceptibility Assessment Based on Novel Neural-Metaheuristic Geographic Information System Based Ensembles

**DOI:** 10.3390/s19214698

**Published:** 2019-10-29

**Authors:** Hossein Moayedi, Abdolreza Osouli, Dieu Tien Bui, Loke Kok Foong

**Affiliations:** 1 Department for Management of Science and Technology Development, Ton Duc Thang University, Ho Chi Minh City, Vietnam; 2 Faculty of Civil Engineering, Ton Duc Thang University, Ho Chi Minh City, Vietnam; 3 Civil Engineering Department, Southern Illinois University, Edwardsville, IL 62026, USA; aosouli@siue.edu; 4 Institute of Research and Development, Duy Tan University, Da Nang 550000, Vietnam; 5 Geographic Information System Group, Department of Business and IT, University of South-Eastern Norway, N-3800 Bø i Telemark, Norway; 6 Faculty of Engineering, Universiti Teknologi Malaysia, 81310 Johor Bahru, Johor, Malaysia

**Keywords:** neural-metaheuristic algorithms, grey wolf optimization (GWO), biogeography-based optimization (BBO), landslide susceptibility mapping

## Abstract

Regular optimization techniques have been widely used in landslide-related problems. This paper outlines two novel optimizations of artificial neural network (ANN) using grey wolf optimization (GWO) and biogeography-based optimization (BBO) metaheuristic algorithms in the Ardabil province, Iran. To this end, these algorithms are synthesized with a multi-layer perceptron (MLP) neural network for optimizing its computational parameters. The used spatial database consists of fourteen landslide conditioning factors, namely elevation, slope aspect, land use, plan curvature, profile curvature, soil type, distance to river, distance to road, distance to fault, rainfall, slope degree, stream power index (SPI), topographic wetness index (TWI) and lithology. 70% of the identified landslides are randomly selected to train the proposed models and the remaining 30% is used to evaluate the accuracy of them. Also, the frequency ratio theory is used to analyze the spatial interaction between the landslide and conditioning factors. Obtained values of area under the receiver operating characteristic curve, as well as mean square error and mean absolute error showed that both GWO and BBO hybrid algorithms could efficiently improve the learning capability of the MLP. Besides, the BBO-based ensemble surpasses other implemented models.

## 1. Introduction

Varnes and Radbruch-Hall [1] described the landslide as all gravity-triggered downward mass movements on slopes. It is one of the most disastrous geohazards which cause huge physical and financial damages worldwide. According to Mihir, et al. [2], the world witnesses around 375 fatal landslides each year, which cause approximately 4600 loss of lives annually. Landslide Working Party of Iran reported that landslide is responsible for the death of around 187 people in this area [3]. Notably, the largest debris flow in the world (Seimareh landslide) has also occurred in Iran [4]. Therefore, having a reliable approximation of landslide susceptibility in the prone areas is a vital prerequisite for land use management as well as future planning.

Landslide susceptibility mapping is an appropriate response to the mentioned demand for alleviating the damages [5]. Yalcin [6] defined the landslide susceptibility as the proneness of the terrain for producing slope failures. Up till now, various models have been proposed for analyzing the susceptibility at different study areas [7,8,9]. Chen, et al. [10] investigated the applicability of some simply-used models, including frequency ratio (FR), weights-of-evidence (WoE) and statistical index (SI) methods for landslide susceptibility mapping in Qianyang County, China. Their achievements showed that the FR produced the most accurate map with 79.40% accuracy, followed by WoE (78.53%) and SI (79.35%). Nicu [11] produced the landslide susceptibility map of an area in Romania by using analytic hierarchy process (AHP), FR and SI models. The findings of this paper revealed the superiority of AHP with around 80% accuracy compared to FR and SI, with around 75% and 61% respective accuracies.

Moreover, the application of artificial intelligence (AI) methods like adaptive neuro-fuzzy inference system (ANFIS) and support vector machine (SVM) has attracted increasing attention to achieve a fast, efficient and inexpensive analysis of landslide susceptibility [12,13,14,15,16,17]. Likewise, many scholars have shown the efficiency of artificial neural network (ANN) for this purpose [18,19]. Pradhan, et al. [20] used a back-propagation multi-layer perceptron (MLP) for landslide susceptibility mapping at three areas of Malaysia, namely Cameron Highland, Penang Island and Selangor. The weights of the landslide conditioning factors were determined from the ANN performance. They performed cross-validation and found that the network developed by data from each region performs better for that region; the accuracy of all developed susceptibility maps was higher than 70%. Bui, et al. [21], compared the capability of five landslide predictive methods including SVM, MLP, radial basis function neural networks (RBF), kernel logistic regression (KLR) and logistic model trees (LMT), in analyzing the risk of landslide in Son La hydropower basin, Vietnam. According to the results, the MLP (90.2% accuracy) outperformed SVM, KLR, RBF and LMT with respective accuracies of 88.7%, 87.9%, 87.1% and 86.1%. Moreover, Pham, et al. [22] evaluated the capability of functional trees (FT), MLP and Naïve Bayes (NB) in the same purpose at the Uttarakhand Area of India. The results showed that, although a close area under the curve (AUC) was obtained for MLP (85%) and FT (84.9%), the first model was introduced as the most successful predictive tool. Besides, the NB presented the lowest accuracy (AUC = 83.8%).

Furthermore, the existing drawbacks of intelligent models such as local minimum and dimension dangers [23] have driven scholars to employ metaheuristic techniques to optimize their performance [24,25,26]. Tien Bui, et al. [27] could successfully find the optimal tuning parameters of the Least-Squares Support Vector Machines (LSSVM) model using differential evolution (DE), for spatial analysis of shallow landslides in Vietnam. Likewise, Tien Bui, et al. [26] presented an improved prediction of landslide susceptibility by synthesizing relevance vector machine (RVM) with imperialist competitive algorithm (ICA). Also, the SVM and logistic regression (LR) were considered as benchmark models. This research showed that the proposed RVM-ICA outperforms SVM and LR with respective AUCs of 92%, 91% and 87%. Jaafari, et al. [25] optimized the performance of ANFIS by using grey wolf optimization (GWO) and biogeography-based optimization (BBO) algorithms. Based on the obtained root mean square error (RMSE) and AUCs (AUCANFIS-BBO = 0.95; RMSEANFIS-BBO = 0.316 vs. ACUANFIS-GWO = 0.94; RMSEANFIS-GWO = 0.322), both applied metaheuristic algorithms performed efficiently for the defined objective. Also, in a research by Moayedi, et al. [28], the membership function parameters of the ANFIS were optimized by four well-known algorithms (particle swarm optimization (PSO), genetic algorithm (GA), ant colony optimization (ACO) and differential evolution (DE)) for analyzing the susceptibility of landslides induced by the Ludian earthquake. As for ANNs, Moayedi, et al. [29] used the PSO for optimizing the MLP neural network for landslide susceptibility modeling at Kermanshah province, western Iran. They concluded that the PSO could be promisingly employed for finding the best computational parameters of the MLP due to higher accuracy of PSO-ANN compared to unreinforced MLP. Similarly, Bui, et al. [30] enhanced the prediction capability of the ANN using Harris hawks optimization (HHO) algorithm in the same field in Kurdistan Province, western Iran.

As is seen, hybrid evolutionary algorithms have been successfully used for optimizing various predictive models. However, in the case of the ANNs, there are still some gaps in knowledge. In this research, it is attempted to present proper optimizations of this tool by using the GWO and BBO evolutionary techniques. The authors came across only a few studies that conduct the optimization of ANNs for landslide susceptibility modeling [29]. As well as this, although some researchers have utilized the GWO and BBO for different aims like flood [31] or forest fire [32] susceptibility mapping, the efficiency of them has not been investigated along with the ANN for landslide susceptibility analysis.

## 2. Study Area

**Figure 1:**
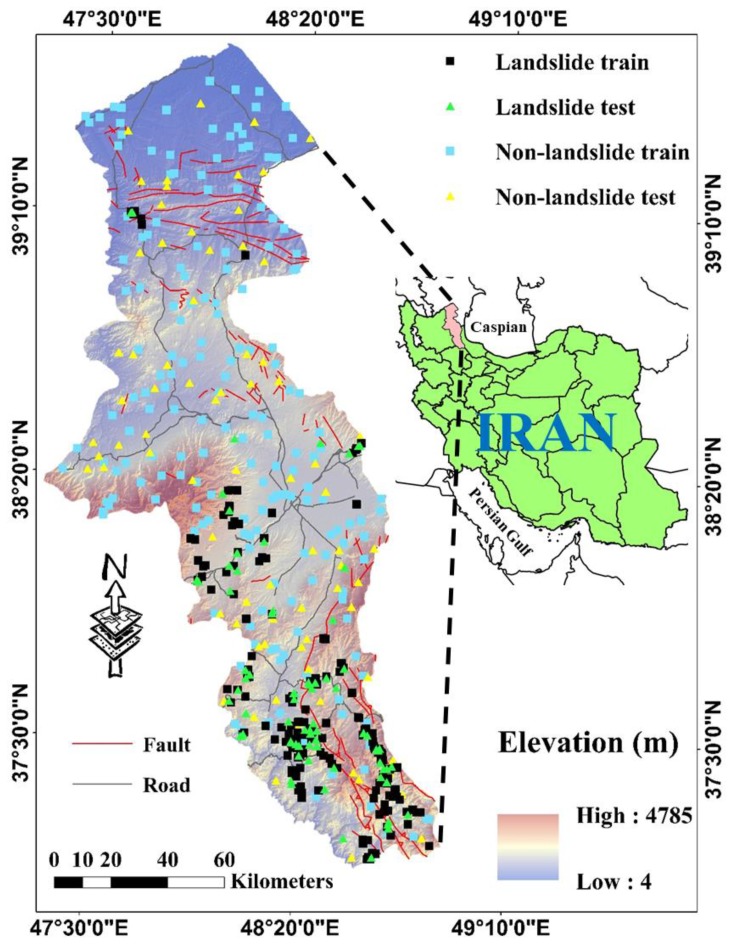
Location of the study area and spatial distribution of the landslides.

Ardabil Province of Iran is selected as the study area. Covering around 17,708 km^2^, it is one of the 31 provinces of Iran located in the vicinity of the Caspian Sea. Figure 1 illustrates the exact location of the study area, which lies within the longitude 47°20′ to 48°60′ E and latitude 37°10′ to 39°40′ N. This Figure shows the location of the marked landslide and non-landslide points as well.

Due to proximity to the sea in the north, as well as Alborz Mountains in the south, the elevation ranges from 4 to 4785, where approximately 40% of the area is higher than 2000 m above the sea level. The average annual rainfall and average annual temperature have been reported as 316 mm and 10.9 °C, respectively. The lands are mainly utilized for agriculture and a small part (approximately one-tenth of the study area) is covered by Beech and Oak forests. Referring to the lithology map, 52 different lithology units form the geology of the Ardabil. Among those, two groups, namely “Andesitic volcanic” and “low-level piedmont fan and valley terrace deposits,” are more prevalent, which cover around 21% and 13% of this area, respectively. Moreover, a so-called soil category “Inceptisols” form approximately 40% of the soil map.

## 3. Data Preparation and Spatial Interaction between the Landslide and Conditioning Parameters

Dividing the used data into independent and dependent parameters, landslide inventory map (i.e., the occurrence or non-occurrence of the landslide) acts as the response variable in implementing machine learning models. It also plays the role of an interactive factor in statistical methods [33]. The inventory map of this study was provided through using the existing records, interpreting the aerial photos, supported by field monitoring using Geographic Information System (GPS) on a 1:25000 scale. Finally, a total of 253 landslide locations were marked. Reasonable selection of non-landslide locations is an important task that should be carefully carried out [34]. Accordingly, 253 non-landslide points were randomly produced within the areas devoid of the landslide [35]. Out of those and using a random selection method, 70% (i.e., 177 landslide and 177 non-landslide events) were considered for the training stage. The remaining 30% (i.e., 76 landslide and 76 non-landslide events) were specified to the testing stage.

**Figure 2:**
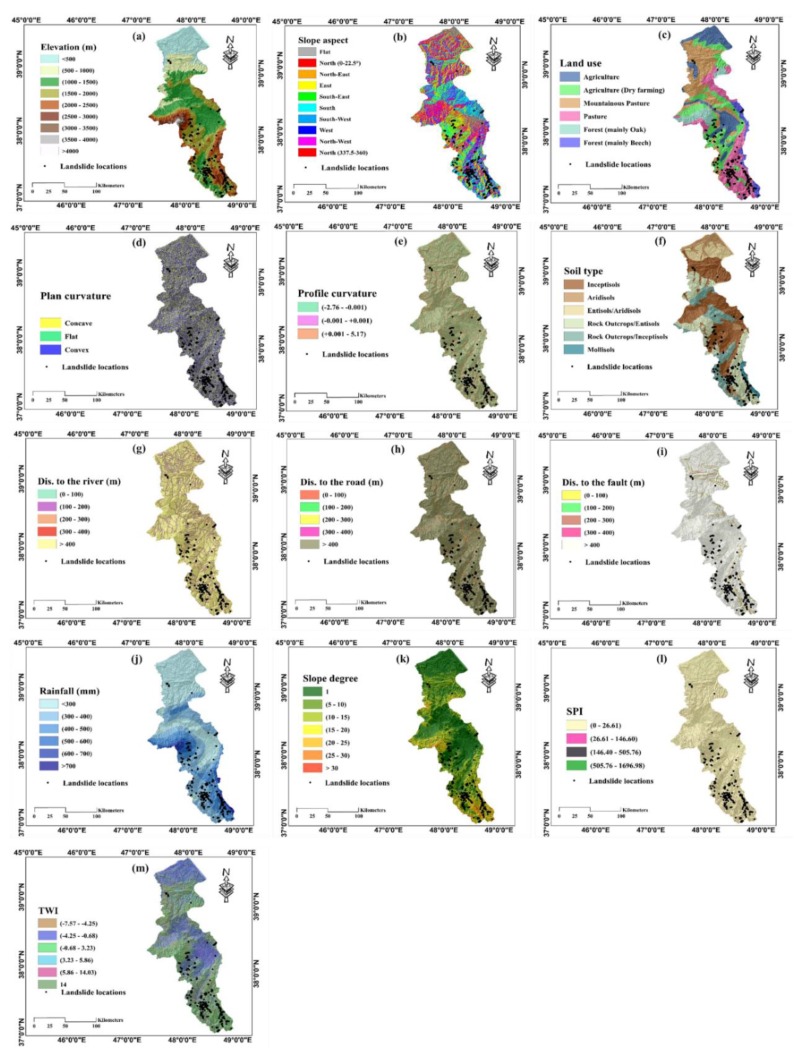
The map of: (**a**) elevation, (**b**) slope aspect, (**c**) land use, (**d**) plan curvature, (**e**) profile curvature, (**f**) soil type, (**g**) distance to river, (**h**) distance from road, (**i**) distance from fault, (**j**) rainfall, (**k**) slope degree, (**l**) stream power index (SPI) and (**m**), topographic wetness index (TWI) landslide conditioning factors.

The independent parameters, that act as the input variables in implementing machine learning models, were selected elevation, slope aspect, land use, plan curvature, profile curvature, soil type, distance to river, distance to road, distance to fault, rainfall, slope degree, stream power index (*SPI*), topographic wetness index (*TWI*) and lithology in this study (Figure 2). Utilizing geographic information system (GIS), the mentioned landslide-related factors were converted to raster from their basic formats, like polygons, polylines, contours and so on.

**Figure 3:**
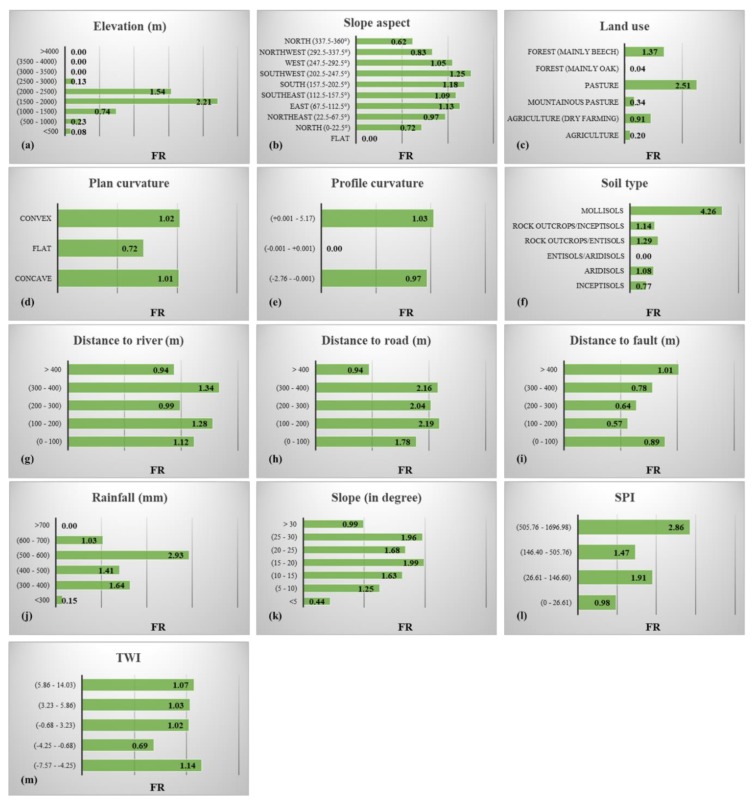
The calculated frequency ratios (FRs) for sub-classes of: (**a**) elevation, (**b**) slope aspect, (**c**) land use, (**d**) plan curvature, (**e**) profile curvature, (**f**) soil type, (**g**) distance to river, (**h**) distance from road, (**i**) distance from fault, (**j**) rainfall, (**k**) slope degree, (**l**) SPI and (**m**) TWI landslide conditioning factors.

The frequency ratio (FR) index was used to explore the spatial interaction between the landslide and conditioning factors. This index gives the ratio of landslide occurrence probability to the non-occurrence probability for a given attribute [36]. Here, it measures the correlation between the landslide and sub-classes of a conditioning factor [37]. For the *FR*, the average value is 1. The values greater than 1 indicate a higher correlation and vice versa. Equation (1) expresses the formulation of the FR.

(1) $$FR=\frac{P_{ls}}{P_{domain}}$$

where, the terms *P_ls_* and *P_domain_* symbolize the percentage of the landslides found in the proposed sub-class and the percentage of the domain covered by it, respectively. Figure 3 presents the calculated value for FR.

The digital elevation model (DEM) of the Ardabil was the source for producing the four other GIS layers, namely, elevation, plan curvature, slope aspect and slope degree. It was created from topographic data (contours and survey base points) on a 1:25,000 scale. As explained earlier, the altitude varies from around 0 to 4785 m. It was divided into nine groups with 500 m intervals [38]. The elevation groups denoting the values between (1500–2000) and (2000–2500) took the largest FR, that is, 2.21 and 1.54, respectively. Regarding the overall range of [0–360°], the slope aspect layer was classified into nine groups, namely Flat, North (0–22.5° and 337.5–360°), North-East (22.5–67.5°), East (67.5–112.5°), South-East (112.5–157.5°), South (157.5–202.5°), South-West (202.5–247.5°), West (247.5–292.5°), North-West (292.5–337.5°) and North (337.5–360°) and the highest correlation with the landslide was obtained for the South-West category. Six different utilizations of land are found with the names “Agriculture,” “Agriculture (dry farming),” “Mountainous Pasture,” “Pasture,” “Forest (mainly Oak),” and “Forest (mainly Beech).” Based on the calculated FR, the fourth group is the most effective factor (FR = 2.51), followed by the last group (FR = 1.37). Examining the plan curvature map, almost equal FRs were obtained for the convex and concave terrains. As for the profile curvature, it was classified into three classes of (−2.76–−0.001), (−0.001–+0.001) and (0.001–5.17) [39]. The analysis of spatial interaction showed that only the third groups took the FR > 1 (i.e., 1.03). Six soil labels were found, namely “Inceptisols,” “Aridisols,” “Entisols/Aridisols,” “Rock Outcrops/Entisols,” “Rock Outcrops/Inceptisols,” and “Mollisols.” The largest FR was obtained for the last group, while it covers only 2% of the study area. Three distance-based layers were produced from the linear phenomena of the rivers, roads and faults. These layers were all classified into five categories with 100 m intervals [19,39]. In this sense, places with (300–400) m distance from the river, (100–200) m distance from the road and (>400) m distance from the faults were found to be the most significant sub-groups. Rainfall map was divided into six extents, namely (<300) mm, (300–400) mm, (400–500) mm, (500–600) mm, (600–700) mm and (>700) mm [19]. The results showed that the values between 3 00 mm and 700 mm have a good correlation with landslide occurrence (FR > 1), where the most correlated is (500–600) mm with an FR close to 3. The slope ranges from 0 to 63°, which are mostly gentle (i.e., slope < 10°). The map was stratified into six groups with 5° intervals [40,41]. The slopes between 1 5° and 30° took the FRs larger than 1.50. Stream power index and topographic wetness index map of the study area were generated by applying Equations (2) and (3), respectively [42,43].

*SPI = α × tanβ* (2)

**Figure 4:**
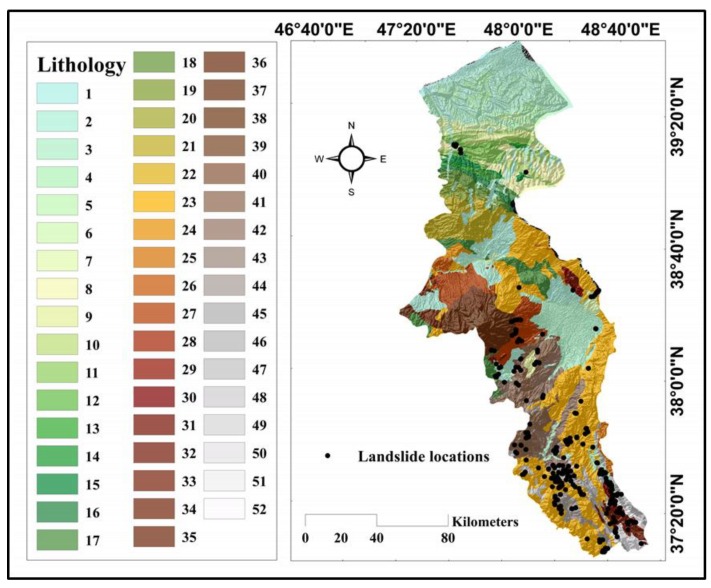
The lithology map of the study area.

*TWI = ln (α/tanβ)* (3)

where the terms *α* and *β* stand for specific catchment and gradient, respectively. As explained supra, 52 different geology units construct the lithology map (Figure 4). Table 1 presents a description of lithological units. According to this table, three lithology units of “Dark grey shale and sandstone (SHEMSHAK FM)” (FR = 7.50) and “Fluvial conglomerate, Piedmont conglomerate and sandstone” (FR = 5.82) and “Dacitic to andesitic volcanic” (FR = 5.36) were found to be the most important units.

## 4. Methodology

**Figure 5:**
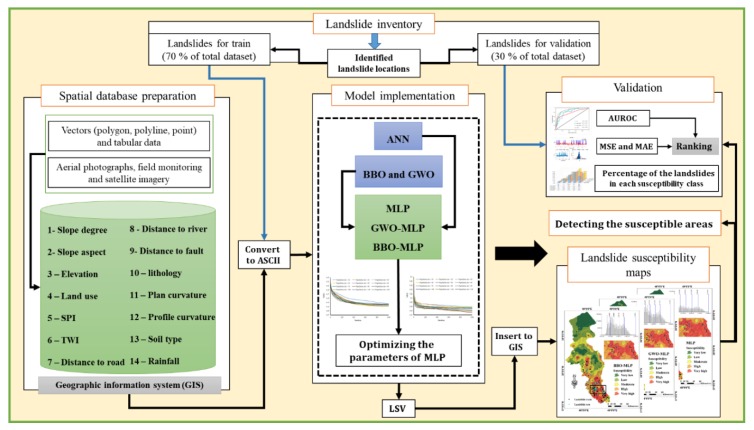
Graphical description of the applied procedure in this study.

To fulfill the purpose of this study, several steps are carried out. These steps are shown in Figure 5. The required GIS layers are produced from their basic formats (i.e., vectors data). Regarding the famous proportion of 70:30%, the landslide inventory map is divided into the training and testing samples, respectively. Then, the values of each landslide conditioning factor are extracted to each landslide and non-landslide point. After providing the proper dataset, the proposed GWO and BBO metaheuristic algorithms are coded to be coupled with the MLP. An extensive trial and error process is proposed to find the most appropriate structure of the GWO-MLP and BBO-MLP. Then, the results of each model are transferred to the GIS environment to produce the landslide susceptibility maps. The accuracy of the obtained maps is evaluated by area under the receiver operating characteristic curve (AUROC). Besides, the error performance of the employed models is measured by means of mean square error (MSE) and mean absolute error (MAE). These criteria are formulated in Equations (4) and (5).

(4) $$\mathrm{MSE}=\frac{1}{N}\overset{N}{\underset{i=1}{\sum }}{\left(Y_{i_{observed}}-Y_{i_{predicted}}\right)}^{2}$$

(5) $$\mathrm{MAE}=\frac{1}{N}\overset{N}{\underset{i=1}{\sum }}\left(Y_{i_{observed}}-Y_{i_{predicted}}\right)$$

in which *N* would be the number of instances and *Yi* observed and *Yi* predicted denote the desired and predicted landslide susceptibility values (LSVs), respectively.

### 4.1. Artificial Neural Network

The name ANN implies a capable neural-based predictive system which can deal with various non-linear engineering problems. Mimicking the biological neural systems, the idea of this model was first suggested by McCulloch and Pitts [44]. Like other machine learning techniques, for developing ANN, two groups of data are required. Firstly, the group which contains the majority of data, called the training dataset, is used for pattern recognition through establishing mathematical equations. Then, the efficiency of the developed network is evaluated by the second group, called testing dataset. Among diverse types of ANNs, MLP is one of the most powerful tools which has been successfully used in many fields of study [45,46,47].

**Figure 6:**
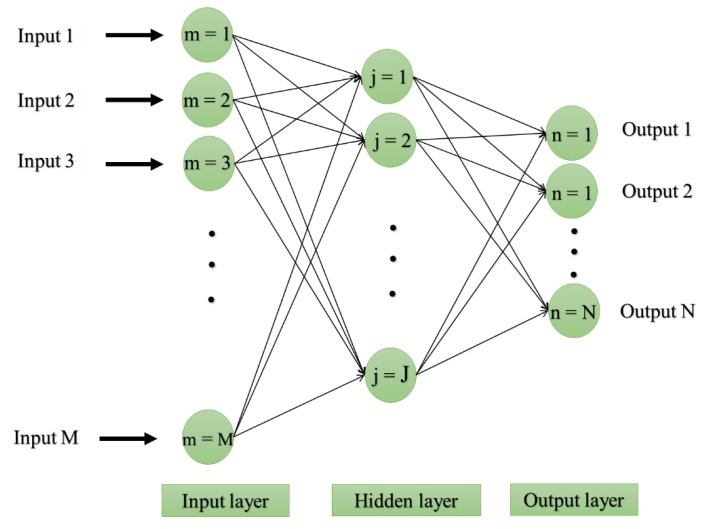
Typical multi-layer perceptron (MLP) structure.

Figure 6 depicts the structure of a typical MLP. Notably, the MLP used in this study uses the Levenberg-Marquardt (LM) training algorithm [48] and backpropagation (BP) learning method [49], due to their excellent performance in previous researches. In the BP method, after accomplishing the training process, the performance error (i.e., the difference between the actual and predicted response variable) is calculated and is propagated back-ward to update the computational weights (W) and biases (b). Let *F*, *T* = {a_i, b_i, c_i …; i = 1, 2, …, K} and *O* = {α_i, β_i, γ_i …; i = 1, 2, …, K} be the activation function, input vectors and output vectors, respectively. Then, the performance of the jth computational units (i.e., neurons) of the MLP can be expressed as follows:

(6) $$O_{j}=F\;(\overset{M}{\underset{m=1}{\sum }}T_{m}W_{mj}+b_{j})$$

### 4.2. Grey Wolf 0ptimization

**Figure 7:**
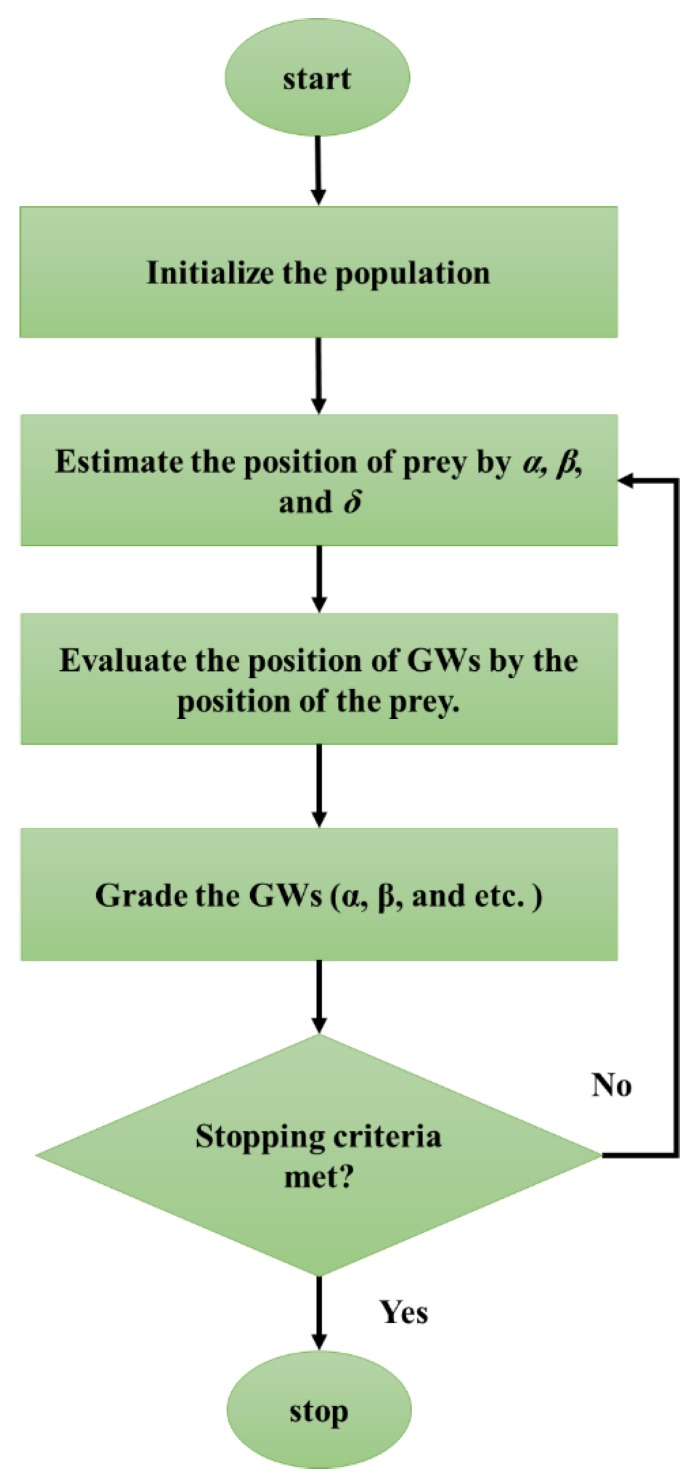
The flowchart of the grey wolf optimization (GWO) algorithm

Mimicking the foraging behavior of grey wolf herds, GWO was presented by Mirjalili, et al. [50]. The GWO is an advanced evolutionary algorithm that follows a social hierarchy. Figure 7 illustrates the flowchart of this technique. The heads of the herd are several male and female wolves, named alpha (*α*), which are decision-makers for the activities like resting and hunting. The next relations are known as beta (*β*) which assist (and obey) the first group for making decisions. Establishing the discipline of the herd is the main duty of the beta wolves. They are also the most appropriate substitutions for the alpha when they retire or die. The next group consists of delta (*δ*), which should act as hunters, sentinels, scouts and so on. The last group, which are also the weakest relations and babysitters, are omega (ω) wolves. Even though they are the weakest wolves, it is likely to observe internal fights without them. The major social behavior of the herd is hunting associated with a social hierarchy [51,52].

Three major steps of the GWO algorithm are (i) detecting, following and approaching the target, (ii) surrounding the target and (iii) attacking the target [53]. The first group (*α*) is considered as the highest-fitted solutions followed by *β*, *δ* and ω. Mathematically, the encircling formula can be expressed as follows:

(7) $$\vec{D}=\left|\;\vec{S}.\;{\vec{P}}_{p}\left(t\right)-\bar{P}\left(t\right)\;\right|$$

(8) $$\vec{P}\left(t+1\right)={\vec{P}}_{p}\left(t\right)-\vec{F}\;.\;\vec{G}$$

where for the iteration time t, $\vec{D}$ is the vector which suggests a new position for a wolf. The terms $\vec{F}$ and $\vec{S}$ are vectors of the coefficient and (${\vec{P}}_{p}$) and $\vec{P}$ denote the position of the prey and wolf, respectively.

Assuming that *α*, *β* and *δ* have more reliable knowledge about the location of the prey, the most promising solutions for these three positions could be registered. Next, other wolves will update the positions accordingly.

(9) $${\vec{D}}_{\alpha }=\left|\;{\vec{S}}_{1}.\;{\vec{P}}_{\alpha }-\vec{P}\;\right|$$

(10) $${\vec{D}}_{\beta }=\left|\;{\vec{S}}_{2}.\;{\vec{P}}_{\beta }-\vec{P}\;\right|$$

(11) $${\vec{D}}_{\delta }=\left|\;{\vec{S}}_{3}.\;{\vec{P}}_{\delta }-\vec{P}\;\right|$$

(12) $${\vec{P}}_{1}={\vec{P}}_{\alpha }-F_{1}.\;{\vec{D}}_{\alpha }$$

(13) $${\vec{P}}_{2}={\vec{P}}_{\beta }-F_{2}.\;{\vec{D}}_{\beta }$$

(14) $${\vec{P}}_{3}={\vec{P}}_{\delta }-F_{3}.\;{\vec{D}}_{\delta }$$

(15) $$\vec{P}\left(t+1\right)=\frac{{\vec{P}}_{1}+{\vec{P}}_{2}+{\vec{P}}_{3}}{3}$$

Eventually, the attack takes place when the target is stopped. This is worth noting that the F contains random variables between −2α and 2α which says the wolves attack the target (|A| < 1) or seek a more suitable one (|A| > 1) [51].

### 4.3. Biogeography-Based Optimization

**Figure 8:**
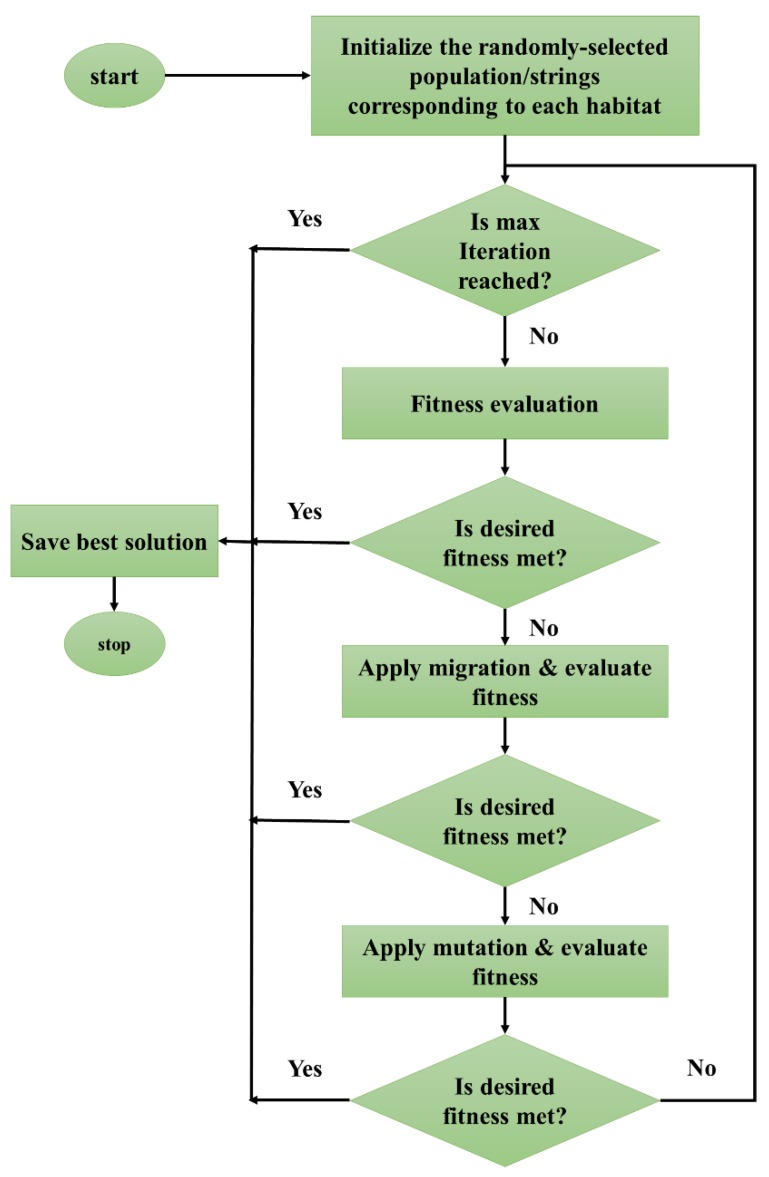
The flowchart of the biogeography-based optimization (BBO) algorithm.

Inspired by the biogeography knowledge and distribution of different species, BBO was proposed by Simon [54]. The BBO is a population-based search technique which was first applied to an MLP by Mirjalili, et al. [55] to optimize its performance. Figure 8 illustrates the flowchart of this algorithm. Like other optimization methods, the BBO gets started by producing a so-called random population “habitat.” These individuals represent possible solutions which are evaluated by habitat suitability index (HSI). Besides, the habitability of the habitats and areas is measured by the suitability index variable (SIV). More clearly, an SIV is a group of real numbers which indicate the population of the candidate solutions. The BBO draws on two major operations, namely migration and mutation.

In the migration, the main aim is to enhance the quality of the possible solutions by modifying them based on other existing solutions. In this step, an immigration rate (*λg*) is defined to decide about the necessity of modification of each SIV [56]. When it is decided to apply the modification, an emigration rate (*μ_g_*) is defined to probabilistically select the solution that will migrate. Notably, like other metaheuristic algorithms, the elite solutions are kept away from this operation to avoid probabilistic corruption [57].

Due to some natural hazards threatening geographical areas, abrupt changes of HSI values can be observed. The habitat probably swerves from its equilibrium HSI. This process is named mutation and its rate is denoted by the probability of each species count. This probability is expressed as follows:

(16) $$P_{g}^{f}=\{\begin{array}{c} -\left(\lambda_{g}+\mu_{g}\right)P_{g}+\mu_{g+1}P_{g+1}\;\;\;\;\;\;\;S=0,\\ -\left(\lambda_{g}+\mu_{g}\right)P_{g}+\lambda_{g-1}P_{g-1}+\mu_{g+1}P_{g+1}\;\;\;\;1\leq S\leq S_{\max}-1,\\ -\left(\lambda_{g}+\mu_{g}\right)P_{g}+\lambda_{g-1}P_{g-1}\;\;\;\;\;\;\;\;\;\;\;\;\;S=S_{\max}. \end{array}$$

in which *S* is the number of species in the habitat.

Each population relation receives a probability to decide the relation will mutate or not. It also denotes the possibility of selecting that relation as the solution to the existing problem. A higher probability value represents that the solution is closer to the overall solution, which means the proposed member should not mutate [58].

## 5. Results and Discussion

This paper addresses two novel optimizations of artificial neural networks for spatial prediction of landslide mapping. To this end, a multi-layer perceptron neural network is synthesized with grey wolf optimization and biogeography-based metaheuristic algorithms for improving its performance. After providing the proper spatial database and pre-processing, the mentioned models are designed in the programming language of MATLAB (on the operating system at 2.5 GHz and six gigs of RAM). Based on the developed codes, in the first step, the computational parameters of the MLP (i.e., the weights and biases) are extracted from the general equation of this model. Then, after determining the appropriate population size, the main loop gets started. It performs within enough repetitions to optimize the received parameters. The results of this process are optimal values of weights and biases. In other words, the main contribution of the used optimization techniques to the existing problem (i.e., landslide susceptibility analysis) is determining the most appropriate computational weights for the landslide conditioning factors. Finally, a new MLP is reconstructed by the optimized parameters to calculate the outputs.

### 5.1. Optimization of the Used Models

**Figure 9:**
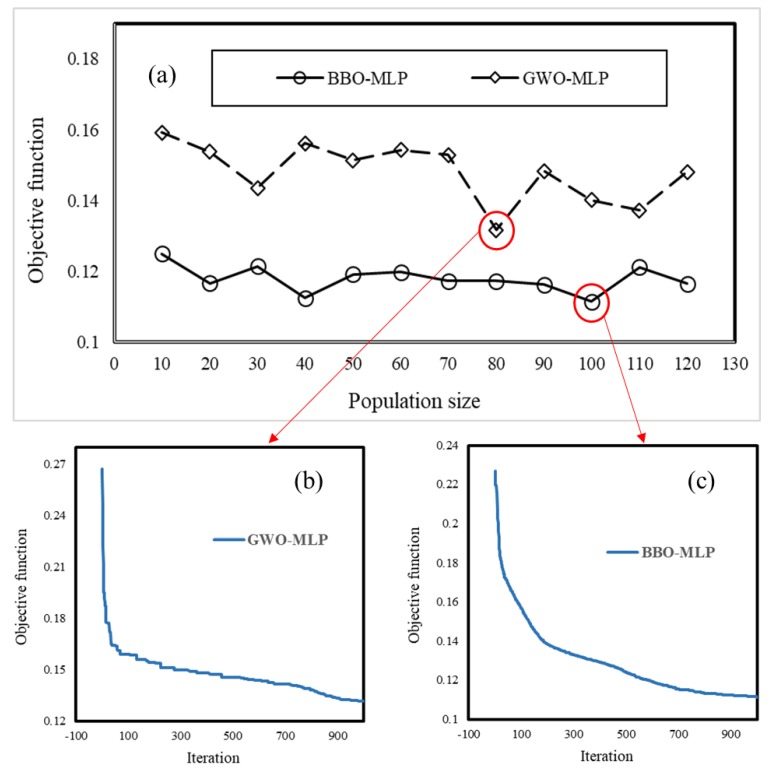
(**a**) The results of the executed sensitivity analysis, (**b**,**c**) the convergence curve of the elite GWO-MLP and BBO-MLP, respectively.

As mentioned previously, the performance of all evolutionary algorithms is evaluated by means of a cost function (CF). It usually represents the error of the produced outputs in each iteration and is projected to decrease over time. In this paper, the MSE is used as the CF. For finding the optimal structure of GWO-MLP and BBO-MLP ensembles, twelve different networks with population sizes of 10, 20, 30, 40, 50, 60, 70, 80, 90, 100, 110 and 120 were tested. Each model performed in 1000 iterations to have enough chance for convergence. Remarkably, to examine the repeatability of the used ensembles, each process repeated five times. The best network was saved and the rest were eliminated. Setting aside the casual results, good overall repeatability was observed for all tested structures. The final results are presented in Figure 9a. The lowest and highest obtained MSEs for the GWO-MLP were 0.1316 and 0.1591, respectively for the population sizes of 80 and 10. As for the BBO-MLP, the highest MSE was similarly obtained for the first population size (MSE = 0.12501). The best-trained network (MSE = 0.11139) was the BBO-MLP developed with population size = 100. Moreover, the convergence curve of the elite networks is shown in Figure 9b,c, respectively for the GWO-MLP and BBO-MLP ensembles.

### 5.2. Susceptibility Maps

**Figure 10:**
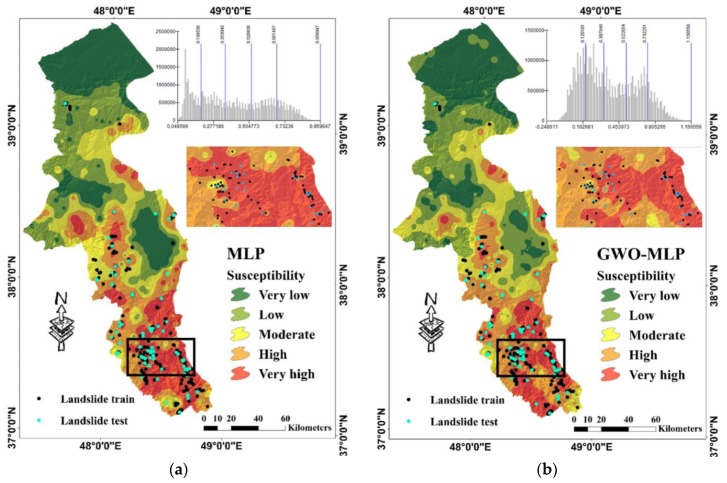

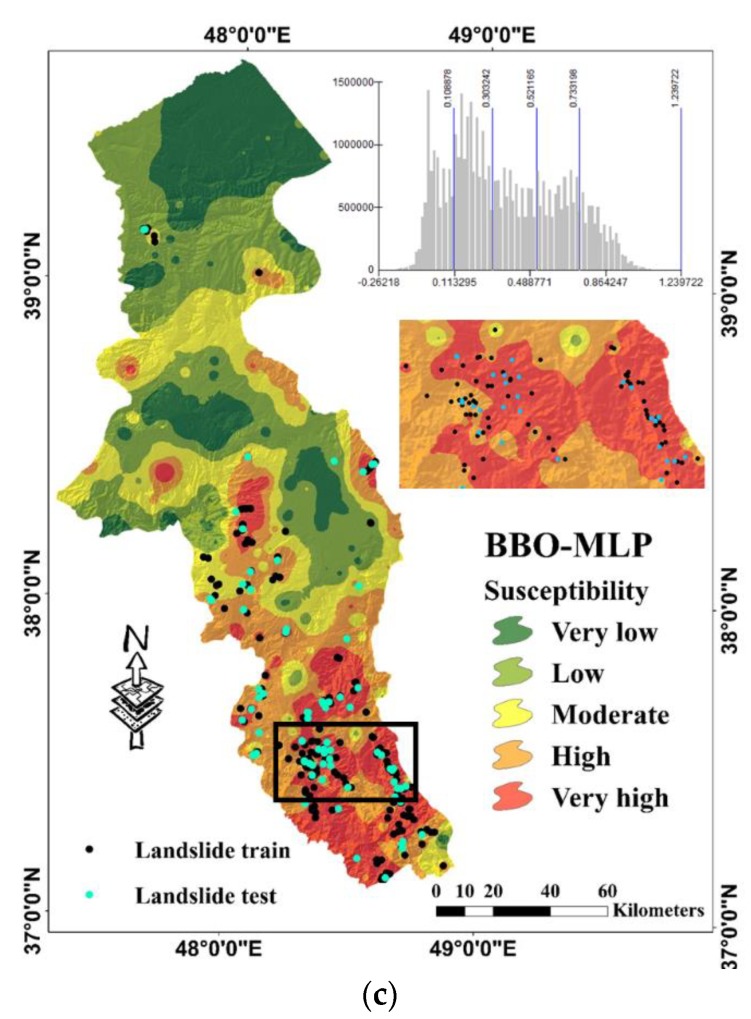
Landslide hazard map produced by (**a**) MLP, (**b**) GWO-MLP and (**c**) BBO-MLP models.

For depicting the landslide susceptibility maps, outputs of all implemented models were extracted and transferred to GIS. The landslide susceptibility values obtained from the MLP, GWO-MLP and BBO-MLP varied in the respective ranges of (0.049599 to 0.959947), (−0.248611 to 1.156556) and (−0.262180 to 1.239722). Then, similar to previous studies [59,60,61,62], each map was classified into five susceptibility categories of “Very Low”, “Low”, “Moderate”, “High” and “Very high”. Figure 10a–c, illustrate the produced landslide susceptibility maps along with the histogram of the outputs for each model. Table 2 presents the percentage of the area which pertains to each susceptibility class. Accordingly, 34.79% (6160.48 km^2^), 32.18% (5699.05 km^2^) and 32.40% (5738.02 km^2^) of the Ardabil province are categorized as highly-susceptible regions, respectively by MLP, GWO-MLP and BBO-MLP. Besides, the mentioned models classified around 8310 km^2^, 8580 km^2^ and 8457 km^2^ of the study area (Total area = 17,708 km^2^) as low and very low landslide susceptibility. Moreover, the ratio of the area belonging to moderate class ranges between 18% and 20% for all three models.

From Table 2, all three models had a good approximation from the location of the occurred landslides. Additionally, all three maps indicate that the southern part of the Ardabil Province is under the dangerous level of susceptibility (i.e., high and very high classes). On the other side, northern parts of Ardabil which are close to the Caspian Sea are recognized as safe areas. Moreover, the landslides detected in the central parts of the study area are mostly located in high and very high susceptibility classes. Focusing on the maps, it can be found that some central regions which are devoid of the landslide, have been classified as dangerous areas that show a high likelihood of landslide occurrence in the future.

**Figure 11:**
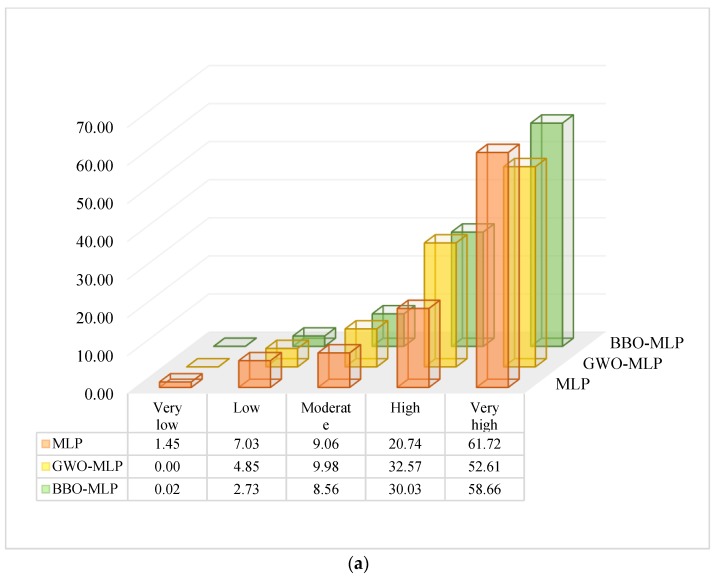

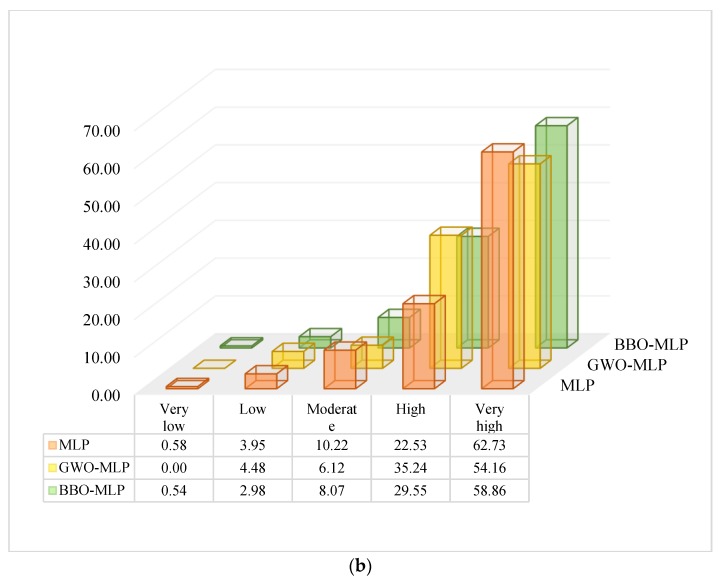
The percentage of the (**a**) training and (**b**) testing landslides located in each susceptibility class.

The percentage of landslides located in each susceptibility class was also calculated. The results are shown in the form of 3D bar charts in Figure 11a,b, respectively for the training and testing landslides. According to these charts, 82.4%, 85.18% and 88.69% of the training points, as well as 85.25%, 89.40% and 88.41% of the testing data, are found in high and very high susceptible areas. Besides, except for the training landslides of the MLP, the ratio of landslide points located in very low susceptible area is lower than 1%.

### 5.3. Validation and Comparison

**Figure 12:**
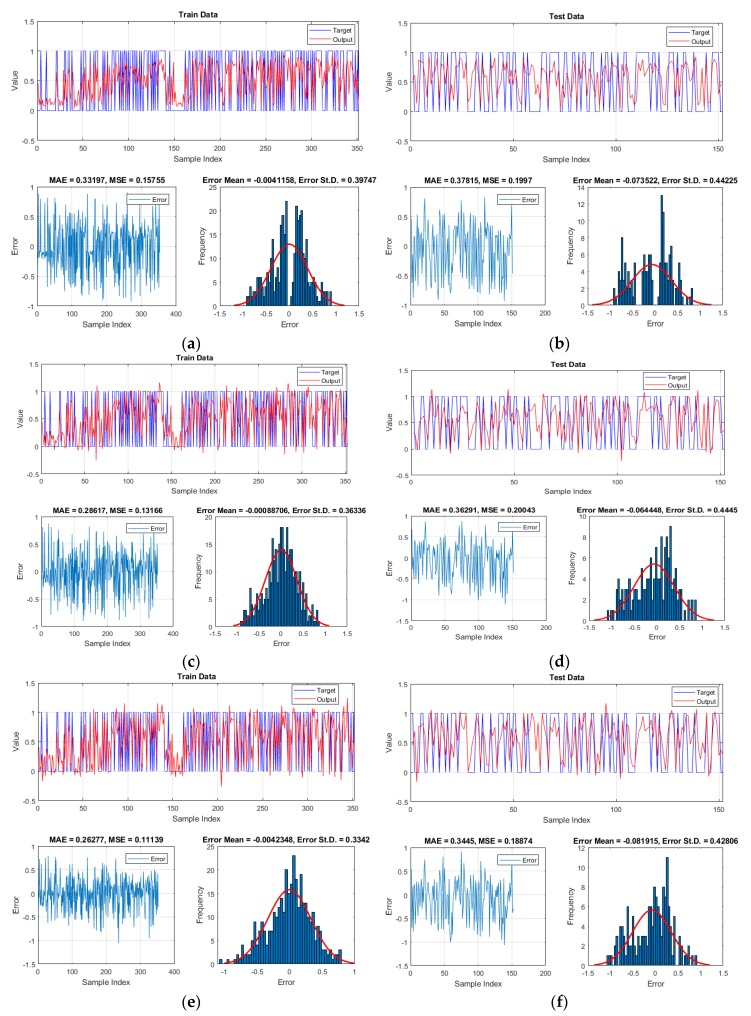
The results obtained for (**a**,**b**) MLP, (**c**,**d**) GWO-MLP and (**e**,**f**) BBO-MLP predictions, respectively for the training and testing samples.

To validate the performance of the used models, we used MAE and MSE error criteria for measuring the error of the results. These criteria were applied to both training and testing samples. A graphical comparison between the actual and calculated LSVs are presented in Figure 12. As well as this, the error (i.e., the difference between the target and output) is calculated and depicted for each sample. The histogram of the errors is also shown. According to these charts, more consistency of the results can be seen for the optimized networks compared to typical MLP. It indicates the efficiency of the used optimization algorithms in training the MLP neural network.

Based on the MSE (0.1575, 0.1316 and 0.1113, respectively for the MLP, GWO-MLP and BBO-MLP) and MAE (0.3319, 0.2861 and 0.2627) obtained for the training samples, training error decreased by 16.44% and 29.33% in term of MSE and by 13.80% and 20.85% in term of MAE, respectively by applying the GWO and BBO evolutionary algorithms. As for the testing phase, the computed MSE (0.1997, 0.2004 and 0.1887), as well as MAE (0.3781, 0.3629 and 0.3445), demonstrate that the more quality of training, the less error of generalization. However, the MSE obtained for unreinforced MLP is slightly lower than GWO-MLP. Considering the effect of the applied optimization techniques, the MAE experienced 4.02% decrease, when the GWO is synthesized. Likewise, applying the BBO reduced the testing MSE and MAE, respectively by 5.50% and 8.88%.

In addition, the accuracy of the generated landslide susceptibility maps was calculated by drawing the receiver operating characteristic (ROC) curves. According to Egan [63], drawing the ROC curve is suitable for measuring the accuracy of diagnostic issues. Also, the area under this curve (AUROC) indicates the accuracy of the proposed prediction. Generally, the AUROC varies from 0.5 to 1, which is directly proportional to the accuracy. In other words, 1 indicates an excellent prediction and adversely, a casual prediction is shown by 0.5.

**Figure 13:**
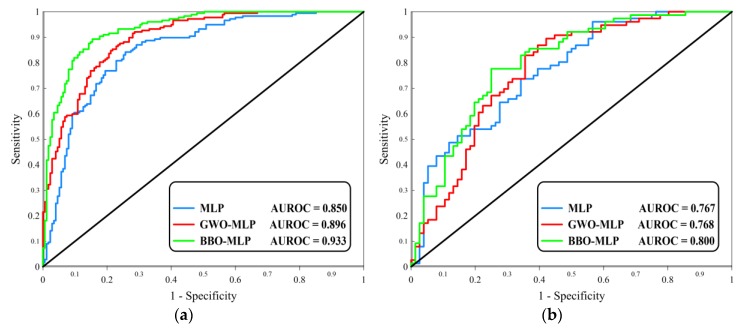
The ROC diagrams plotted for the (**a**) training and (**b**) testing data.

Figure 13 shows the plotted ROC curves for MLP, GWO-MLP and BBO-MLP results in training and testing phases. The AUROC was computed and the results are in a good agreement with the MSE and MAE indices. Due to a significant rise in the MLP accuracy from 85.0% to 89.6% and 93.3%, it can be deduced that the GWO and BBO optimization algorithms have performed efficiently in finding the optimal weights and biases of MLP for analyzing the landslide susceptibility in the proposed area. Also, testing results attest to the usefulness of the BBO-algorithm. However, similar to testing MSE, there was a close competition between the generalization power of the MLP and GWO-MLP. In this part, the AUROCs were obtained 0.767 and 0.768, respectively.

Table 3 summarizes the obtained values of three used accuracy criteria for both training and testing landslides. Based on this table, it is deduced that in comparison with the GWO products, the weights and biases that are suggested by the BBO construct a more accurate MLP neural network. Moreover, considering the calculated AUROCs, the susceptibility map developed by this model is more reliable.

In the last part of this section, it was aimed to present an accurate yet inexpensive and simple solution for predicting the LSV, which is a non-linear and complex problem. Note that, the conditioning factors considered in this study were elevation, slope aspect, land use, plan curvature, profile curvature, soil type, distance to the river, distance to the road, distance to the fault, rainfall, slope degree, SPI, TWI and lithology. For this purpose, the ruling formula of the most promising predictive model of this study (i.e., the BBO-MLP) was extracted and presented Equation (17).

*LSV = −0.3868 × Z1 + 0.1195 × Z2 + 0.0537 × Z3 − 0.8621 × Z4 + 0.0356 × Z5 + 0.6738* (17)

where,

*Zi = Tansig (Wi1 × elevation + Wi2 × slope aspect + Wi3 × land use + Wi4 × plan curvature + Wi5 × profile curvature + Wi6 × soil type + Wi7 × distance to the river + Wi8 × distance to the road + Wi9 × distance to the fault + Wi10 × rainfall + Wi11 × slope degree + Wi12 × SPI + Wi13 × TWI + Wi14 × lithology + bi)* (18)

in which, the term *Tansig* represents the MLP activation function which for an input x is expressed as follows:

(19) $$Tansig\left(x\right)=\frac{2}{1+e^{-2x}}-1$$

Also, *Wi1*, *Wi2*, …, *Wi14* and bi are the computational weights and biases of the MLP which were optimized by the BBO algorithm. These parameters are available in Table 4.

## 6. Discussion

Having a reliable approximation of landslide susceptibility is a significant prerequisite for engineers and land-use planners. One of the main objectives of this task is detecting highly-susceptible regions (i.e., over a specific area) to adopt future decisions for alleviating the damages triggered by this natural hazard. Various predictive and evaluative techniques have been successfully developed to deal with the spatial prediction of the landslide. Due to recent advances, novel intelligent models have attracted great attention for this purpose. High robustness of artificial neural networks has been confirmed in the spatial assessment of different phenomena including landslide [20,40,64], flood [65] and gully erosion [66]. Pradhan and Lee [67] successfully used a multi-layer perceptron neural network for producing the landslide susceptibility map of Cameron Highland (Malaysia). They used a back-propagation training method to determine the weight of each causative factor.

Furthermore, many scholars have suggested synthesizing landslide predictive models with metaheuristic optimization algorithms, like genetic algorithm particle swarm optimization and imperialist competition algorithm, in order to prevail the existing drawbacks (e.g., getting trapped in local minima) [26,68]. Moayedi, et al. [29] improved the performance of an MLP neural network by applying the PSO evolutionary algorithms for landslide susceptibility analysis at Layleh valley of Kermanshah, west of Iran. They used twelve landslide conditioning factors, namely elevation, soil type, slope aspect, land use, slope degree, lithology, plan curvature, distance to the road, distance to the river, distance to the fault, SPI and TWI to create the spatial database. As a result of this optimization, the RMSE decreased from 0.1110 to 0.0389.

In the current paper, two wise optimization techniques, namely grey wolf optimization and biogeography-based optimization were applied to a back-propagation-based MLP neural network for optimizing the computational parameters. Similar to other evolutionary algorithms (e.g., PSO, GA, DE, ACO, etc.) the GWO and BBO are population-based search techniques which their advantages over the mentioned optimization techniques are shown in some studies (Reihanian, et al. [69] and references therein). Bui [32] employed the GWO and BBO as well as gravitational search algorithm (GSA) for optimizing the performance of the MLP for investigating forest fire susceptibility in a fire-prone area of Vietnam. The results revealed the efficiency of the GWO and BBO with respective AUCs of 0.9515 and 0.9509. Also, Jaafari, et al. [25] used these algorithms for optimizing a fuzzy system for landslide susceptibility mapping in India. Referring to the calculated RMSEs (0.316 and 0.322, respectively for BBO-ANFIS and GWO-ANFIS) and AUCs (0.95 and 0.94), it was shown that the BBO-based ensemble shows a slightly higher accuracy.

In this paper, although close competition was observed between the MLP (AUROC = 0.767) and GWO-MLP (AUROC = 0.768) for predicting unseen landslides, both GWO and BBO performed efficiently in improving the learning capability of the MLP. In this sense, the training AUROC increased from 0.850 to 0.896 and 0.933, respectively. The findings are in good agreement with similar previous studies. However, these researches are conducted for different areas and also, there are various parameters that influence the performance of the models (e.g., the type and structure of the networks, considered landslide causative factors, proportion of the training and testing data, etc.). For instance, utilizing hybrid methods by Nguyen, et al. [70] and Li, et al. [71] resulted in the improvement of the MLP. By comparison, the superiority of the used BBO can be demonstrated to some state of the art algorithms like the HHO employed by Bui, et al. [30].

Examining the results of the applied trial and error process showed that the BBO constructed a better MLP. However, it needed a larger population (25% larger than what GWO needs). Hence, although utilizing a larger population size may lead to a more complex solution and probably longer calculations, it seems reasonable when the accuracy is a determinant factor. Meanwhile, as a limitation of the study, except for the population size, default values were considered for other parameters of the algorithms. Focusing on the limitation of the BBO, although this algorithm has good exploitation for global optimization, it is slow exploring of the search space [72]. In this sense, it is felt that optimizing the landslide-related factors as well as the parameters of the algorithms can be a proper idea for conducting future studies.

A neural-based mathematical formula was presented to calculate the LSV with considering the used landslide causative factors. It is proper to note that, for many other applications, the outputs of such formulas must be transferred to the real extent. This is because the ANN normalizes the input and target variables during calculations. However, due to the fact that a landslide susceptibility map can be generated by any extent of data, the present formula can be directly used for this aim.

Last but not least, the authors believe that establishing state-of-the-art systems for landslide early warning and monitoring in highly susceptible regions and particularly for critical slopes, will be helpful for diminishing the damages caused by this environmental threat. According to Uchimura, et al. [73] utilizing monitoring and early warning systems are one of the most promising ways for the purpose of landslide risk prevention. Sensors are the main gadgets for this process which analyze the movements of masses to detect and analyze the occurrence of landslide [74,75,76,77]. More information about this argument is detailed in relevant studies [74,75,76].

## 7. Conclusions and Remarks

Due to the wide application of typical optimization techniques like PSO and GA for landslide susceptibility mapping, the main motivation of this paper was employing two wise metaheuristic algorithms of GWO and BBO for this purpose. These algorithms were successfully synthesized with an artificial neural network to optimize its computational parameters. The most appropriate structure of each model was determined through an extensive trial and error process. The results showed that the GWO with population size = 80 and the BBO with population size = 100 yields the best optimization of the MLP. Referring to the results, both used algorithms performed efficiently in improving the learning capability of the MLP. It should be noted that, although applying the BBO lead to a considerable increase prediction capability of the MLP, close accuracies were obtained for the MLP and GWO-MLP. From the comparison viewpoint, the BBO-MLP outperformed GWO-MLP in both training and testing stages. Based on the excellent performance of the developed BBO-MLP model, a neural-based mathematical formula was presented to be used for predicting the LSV in the same environmental and geographical circumstances.

## Figures and Tables

**Table 1:** Description of the lithological units.

Lithology Unit	Description	FR	Lithology Unit	Description	FR
1	Stream channel, braided channel and flood plain deposites	0.00	27	Coarse grained fanglomerate composed of volcaniclastic materials locally with intercalation of lava flows (Lahar)	1.14
2	High level piedmont fan and vally terrace deposits	0.24	28	Gypsiferous marl	0.00
3	Low level piedment fan and vally terrace deposits	0.35	29	Andesitic tuff	0.00
4	Silty clay, sandy tuff and fresh water limestone (Baku Fm)	0.00	30	Light grey, thin-bedded to massive limestone (LAR Fm)	4.39
5	Silty clay, sand, gravel and volcanic ash (Absheran Fm)	0.00	31	Conglomerate and sandstone	0.00
6	Varigated gypsiferous clay shale; conglomerate and sandstone	0.00	32	Pliocene andesitic subvolcanics	0.00
7	Polymictic conglomerate and sandstone	1.54	33	Dark grey shale and sandstone (SHEMSHAK Fm)	7.50
8	Alternation of varigated siltyclay shale with sandstone	0.49	34	Dolomite and sandstone (Bayandour Fm)	0.05
9	Red marl, gypsiferous marl, sandstone and conglomerate (Upper red Fm.)	1.38	35	Granite to diorite	0.00
10	Massive to thick bedded tuffaceous sandstone and varigated shale	0.32	36	Rhyolitic to rhyodacitic tuff	0.00
11	Alternation of sandstone with siltstone and claystone	0.50	37	Andesite to basaltic volcanics	0.65
12	Alternations of marl, silty clay shale, sandstone and dolomitic limestone	1.34	38	Andesitic subvolcanic	0.00
13	sandstone, calcareous sandstone and limestone	0.00	39	Rhyolitic to rhyodacitic volcanic tuff	0.00
14	Red Beds composed of red conglomerate, sandstone, marl, gypsiferous marl and gypsum	0.00	40	Teravertine	0.85
15	Basal conglomerate and sandstone	0.00	41	Dacitic to andesitic subvolcanic rocks	2.31
16	Silty shale, marl, thin-bedded limestone, tuffaceous sandstone and basaltic volcanic rocks	0.00	42	Marl, shale, sandstone and conglomerate	1.77
17	Basaltic volcanic rocks	0.23	43	Andesitic and basaltic volcanics	0.00
18	Silty shale, sandstone, marl, sandy limestone, limestone and conglomerate	0.00	44	Marl, calcareous sandstone, sandy limestone and minor conglomerate	2.70
19	Flysch turbidite, sandstone and calcareous mudstone	0.00	45	sandy to silty gluconitic limestone and calcareous limestone (Shal Fm)	0.68
20	Basaltic volcanic	0.00	46	Fluvial conglomerate, Piedmont conglomerate and sandstone.	5.82
21	Andesitic volcanic	0.00	47	Red and green silty, gypsiferous marl, sandstone and gypsum (Lower Red Fm)	3.04
22	Low-grade, regional metamorphic rocks (Green Schist Facies)	0.00	48	Cretaceous rocks ingeneral	0.82
23	Andesitic volcanics	1.41	49	Dacitic to andesitic volcanic	5.36
24	Dacitic to Andesitic tuff	0.00	50	Gneiss, anatectic granite, amphibolite, kyanite, staurolite schist, quartzite and minor marble (Barreh Koshan Complex and Rutchan Complex)	1.85
25	Upper cretaceous, undifferentiated rocks	0.00	51	Andesitic basaltic volcanic	5.09
26	Andesitic volcanic tuff	0.00	52	Massive grey to black limestone	3.58

**Table 2:** The ratio and area of each susceptibility class.

Susceptibility Class	MLP		GWO-MLP		BBO-MLP	
	Ratio (%)	Area (km*^2^*)	Ratio (%)	Area (km^2^)	Ratio (%)	Area (km^2^)
Very low	24.38	4317.18	23.39	4142.80	18.96	3357.75
Low	22.55	3992.97	25.06	4436.87	28.80	5099.14
Moderate	18.28	3237.48	19.37	3429.38	19.84	3513.20
High	19.06	3374.70	20.24	3584.99	19.34	3425.24
Very high	15.73	2785.78	11.94	2114.06	13.06	2312.78

**Table 3:** The obtained accuracy criteria in the training and testing phases.

Ensemble Models	Network Results					
	Training Phase			Testing Phase		
	MSE	MAE	AUROC	MSE	MAE	AUROC
**MLP**	0.1575	0.3319	0.850	0.1997	0.3781	0.767
**GWO-MLP**	0.1316	0.2861	−0.896	0.2004	0.3629	0.768
**BBO-MLP**	0.1113	0.2627	−0.933	0.1887	0.3445	0.800

**Table 4:** The computational weights and biases of the MLP optimized by BBO algorithm.

*i*	*Wi1*	*Wi2*	*Wi3*	*Wi4*	*Wi5*	*Wi6*	*Wi7*	*Wi8*	*Wi9*	*Wi10*	*Wi11*	*Wi12*	*Wi13*	*Wi14*	*bi*
1	0.3068	−0.3699	−0.2489	0.1434	−0.5437	−0.7416	0.0732	0.0473	−0.1541	−0.0666	−0.7739	0.5818	0.4335	−0.3827	−1.5706
2	0.4424	0.3283	−0.5875	0.4642	−0.5592	−0.5253	0.3199	0.2965	−0.5317	0.3238	−0.5168	−0.3437	0.1962	0.1108	−0.7853
3	−0.4365	0.4556	−0.5879	0.0676	−0.5329	0.3528	0.1440	0.1758	0.1744	−0.0691	0.6151	−0.7576	−0.2494	0.4567	0.0000
4	0.1483	−0.1693	−0.0301	−0.0738	0.4592	−0.7367	−0.2852	0.3932	−0.6592	−0.2104	−0.0276	0.7537	−0.6050	−0.0807	−0.7853
5	−0.5482	−0.5329	−0.5605	−0.2488	0.5488	0.3826	−0.4658	0.1531	0.0340	−0.3164	−0.5746	0.2770	−0.2494	0.4976	−1.5706
